# Motion-Correction Enabled Ultra-High Resolution In-Vivo 7T-MRI of the Brain

**DOI:** 10.1371/journal.pone.0154974

**Published:** 2016-05-09

**Authors:** Christian Federau, Daniel Gallichan

**Affiliations:** 1 Dept of Radiology, Section of Neuroradiology, Stanford University, Stanford, California, United States of America; 2 CIBM, EPFL, Lausanne, Switzerland; National Taiwan University, TAIWAN

## Abstract

**Objectives:**

To demonstrate the image quality that can be obtained for multiple contrasts using ultra-high resolution MRI (highest nominal resolution: 350 μm isotropic) at 7T using appropriate motion-correction.

**Materials and Methods:**

An MRI-based fat-excitation motion navigator (which requires no additional hardware) was incorporated into T1-weighted (MP2RAGE, 350 μm nominal isotropic resolution, total scan time 124 mins over 2 sessions. The MP2RAGE also provides quantitative T1-maps), 3D-TSE (380 μm nominal isotropic resolution, total scan time 58 mins) and T2*-weighted protocols (3D-GRE, 380 μm nominal isotropic resolution, total scan time 42 mins) on a 7T MR system. Images from each contrast are presented from a single healthy adult male volunteer (34 years) for direct comparison. The subject provided written consent in accordance with the local review board.

**Results:**

Images of various brain structures are revealed at unprecedented quality for in-vivo MRI. The presented images permit, for example, to delimit the internal structure of the basal ganglia and thalamus. The single digitationes of the hippocampus are visible, and the gyrus dentatus can be visualized. Intracortical contrast was also observed in the neocortex, including the stria of Gennari of the primary visual cortex.

**Conclusions:**

Appropriate motion-correction allows MRI scans to be performed with extended scan times enabling exceptionally high resolution scans with high image quality, with the use of a 7T scanner allowing large brain coverage for 350–380 μm isotropic voxels with total scan times for each contrast ranging from 42 to 124 minutes.

## Introduction

The maximum achievable resolution of an MRI scan is in principle limited only by the time available to scan the subject, as longer scan times are necessary to compensate for the loss of signal-to-noise ratio (SNR) associated with smaller voxels. Recent technological advancements in both hardware and software have allowed MRI to reach increasingly high spatial resolutions, leading to exquisite images of the brain and enabling in-vivo visualization of structures such as the stria of Gennari, the brainstem[1] and sub-structures of the hippocampus[2,3]. However, a smaller voxel size not only makes the image more susceptible to artifacts associated with small involuntary motion of the subject during the scan, but an extended scan time also means that the total subject-motion is likely to be greater. It has recently been shown that a suitable motion-tracking and correction system allows substantial improvements in image quality for very high resolution imaging, either using a camera-and-marker system [4–6] or additional interleaved lower-resolution MR image ‘navigators’ [7,8]–even when the subjects being scanned are healthy adults accustomed to the scanner environment.

For imaging at the highest resolutions, higher magnetic field strengths are desirable in order to maximize the available SNR. In this work we demonstrate that ultra-high resolution motion-corrected scans can be achieved on a 7T MRI scanner without requiring any additional hardware, delivering images of the whole brain at exceptional quality for various image contrasts.

## Materials and Methods

All data were acquired on a non-clinical 7T head-only MR system (Siemens Healthcare, Erlangen, Germany) fitted with a 32-channel RF coil array housed within a birdcage transmit coil (Nova Medical Inc., Wilmington, MA). The volunteer was a healthy adult male (34 years) who gave informed consent prior to the imaging in accordance with the approval by the local review board (Commission cantonale d’éthique de la recherche sur l’être humain, Vaud. Protocol number 347/14). All images were acquired using isotropic voxel sizes, as this allows a more accurate representation of the anatomy as well as permitting the reformatting of the images along arbitrary slices to better visualize various anatomical features of interest. Isotropic voxels are also more suitable for use in quantitative software analysis such as estimating the variation of MR parameters across cortical layers.

We integrated the fat-based motion-navigators (3D FatNavs) and retrospective motion-correction [8] with three imaging sequences commonly used for ultra-high field MRI: T_1_-weighted MP2RAGE [9] (which gives a uniform, bias-field corrected T_1_-weighted image (MP2RAGE UNI) as well as a quantitative T_1_ map (MP2RAGE T1)), 3D-TSE (Turbo Spin Echo)[10] and T_2_*-weighted 3D-GRE (Gradient-Recalled Echo). Only the GRE scan required additional scan time to integrate the motion-navigators. Parameters for each scan were then selected to achieve ultra-high resolution scans (350–380 μm isotropic voxels)–with the 350 μm MP2RAGE scan repeated over two 1-hour sessions. This was to demonstrate that with robust motion-correction multiple scan sessions can be effectively combined–meaning that particularly high resolution scans need not be restricted by the ~1 hour scan duration limit typically considered to avoid excessive discomfort for the subject.

### 3D FatNavs and motion-correction

The 3D FatNavs used as a motion-navigator consisted of a 3D gradient-recalled echo (GRE) sequence with a three-pulse binomial excitation to selectively excite at the frequency of fat, 2 mm isotropic resolution, 88×128×128 matrix size, echo time (TE) = 1.35 ms, repetition time (TR) = 3.0 ms, bandwidth = 1950 Hz/pixel, flip angle = 7° and ¾ partial Fourier (reconstructed with zero-filling) in both phase-encoding directions. With 4×4 GRAPPA (generalized autocalibrating partially parallel acquisitions[11]) undersampling this requires 1152 ms per acquisition, plus an additional 2.3 s at the start of the scan for the GRAPPA calibration data.

Reconstructed 3D FatNavs were co-registered using the ‘realign’ tool in SPM (Statistical Parametric Mapping, version 8) and the resulting estimated motion used to perform retrospective motion-correction separately for the raw k-space data from each RF channel of the respective host sequence, accounting for rotations by using the 3D non-uniform fast Fourier transform algorithm [12] implemented by Jeffrey Fessler’s reconstruction toolbox (http://web.eecs.umich.edu/~fessler/code/). Assuming small motion, no density compensation was applied. As a final post-processing step, the motion-corrected TSE and GRE data were bias-field corrected by dividing the whole volume by a smooth volume generated by fitting a smooth 3D function to data above a manually chosen threshold using Damien Garcia’s ‘smoothn’ tool[13] (http://www.biomecardio.com/matlab/smoothn.html).

### MP2RAGE data

The MP2RAGE sequence (Magnetization Prepared 2 Rapid Acquisition Gradient Echoes)[9] is an extension to the widely used 3D MPRAGE sequence[14] which combines two GRE volumes acquired at separate inversion times following an adiabatic inversion pulse resulting in a T1-weighted image with spatially normalized contrast–and can be converted to a quantitative map of T1 estimates via a look-up table. We used a nominal isotropic resolution of 350 μm with a matrix size of 552×442×416, inversion times of 800 ms and 2700 ms with flip angles of 5° and 7° during the respective readout trains (echo spacing 7.8 ms), bandwidth = 240 Hz/pixel, ¾ partial Fourier undersampling in both phase encoding directions (reconstructed using zero-filling) and 6000 ms between consecutive inversion pulses. A 3D FatNav was acquired in the available ‘dead-time’ between each second readout train and the next inversion pulse. 2× GRAPPA acceleration was used in the first phase-encoding direction to reduce the readout train duration sufficiently to maintain the desired inversion times, resulting in a total scan duration of 31 minutes for a single scan. A total of 4 scans were acquired over two scanning sessions, each of which was motion-corrected using the 3D FatNavs as described above, and then additionally corrected for distortions due to gradient non-linearities using ‘gradunwarp’ software (https://github.com/ksubramz/gradunwarp) making use of a look-up table of spherical harmonics characterizing the expected distortions which was provided by the scanner manufacturer. Individual motion and distortion corrected volumes were then co-registered using rigid-body alignment with FLIRT software [15] (part of FSL, http://fsl.fmrib.ox.ac.uk) prior to averaging.

To allow visual comparison of the anatomical details that can be resolved at different image resolutions, the final combined 350 μm image was downsampled (also using FLIRT software) to 500 μm and 1 mm isotropic resolution.

### TSE data

A vendor-supplied implementation of turbo-spin echo (TSE) with a variable flip angle train (based on the scheme described by Mugler et al[10]) was used with a nominal isotropic resolution of 380 μm, matrix size = 512×320×448, ¾ partial Fourier undersampling (reconstructed with zero-filling) was used in both phase-encoding directions, TE/TR = 381/2700 ms, Turbo factor = 166, echo train duration = 997 ms, bandwidth = 296 Hz/pixel. The 3D FatNav was acquired between each readout train. This results in a total scan time of 29 minutes for a single scan, which was repeated twice within a single scan session. Motion-correction was performed separately on the two volumes before alignment using rigid-body registration (FLIRT, as for MP2RAGE) and averaging. The manual segmentation of the hippocampus was performed using ITK-SNAP software (http://www.itksnap.org/) and rendered using Blender software (http://www.blender.org).

### GRE data

A nominal isotropic resolution of 350 μm was used with a matrix size of 512×444×320, TE/TR = 15.6/27 ms, ¾ partial Fourier undersampling (reconstructed with zero-filling) in both phase-encoding directions, flip angle = 11°, bandwidth = 60 Hz/pixel. A 3D FatNav was inserted following completion of reach partition-encoding loop, resulting in a total scan time of 42 minutes (which would have been possible in 36 minutes without the addition of the 3D FatNavs acquired every 7.6 s).

## Results

**Fig 1:**
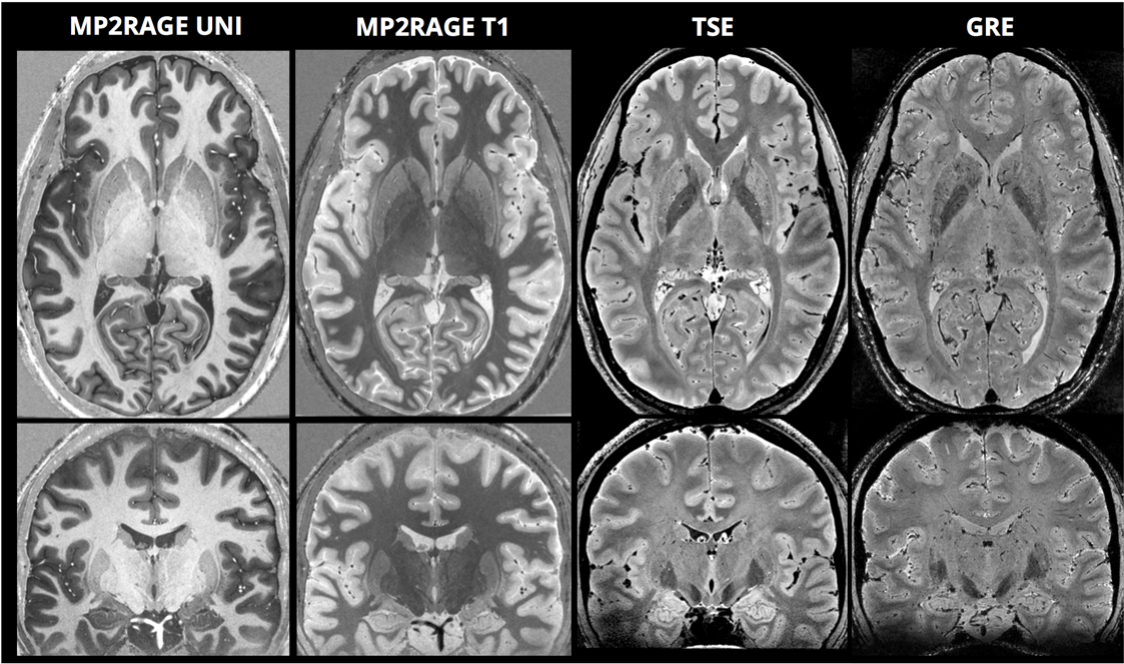
Overview axial and coronal sections for each of the four contrasts: uniform T_1_-weighted image (MP2RAGE UNI), quantitative T_1_ map (MP2RAGE T1), Turbo-Spin Echo (TSE), and Gradient-Recalled Echo (GRE).

**Fig 2:**
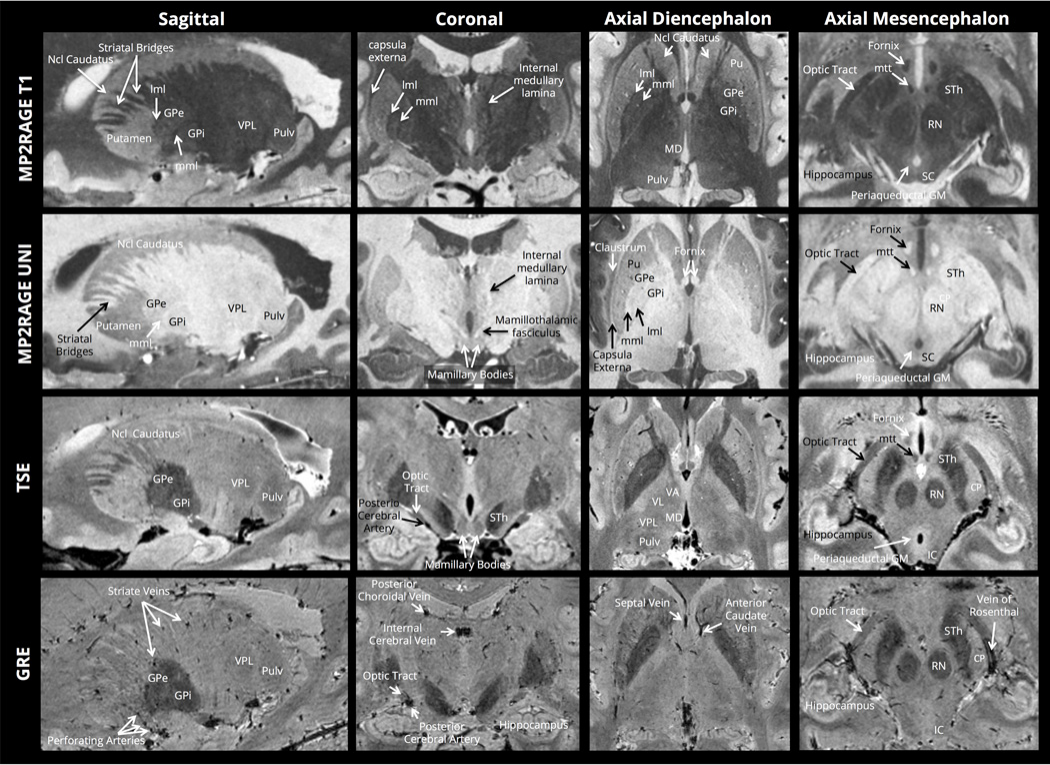
Zoomed sections of the basal ganglia and middle-brain for each of the four ultra-high resolution contrasts; mml: medial medullary lamina; mtt: mamillothalamic tract; lml: lateral medullary lamina; GPe: Globus Pallidus externus; Gpi: Globus Pallidus internus; Pulv: Pulvinar; STh: Sub-Thalamic nucleus; VPL: Ventral Posterior Lateral nucleus; VL: ventral lateral nucleus; VA: Ventral Anterior nucleus; MD Mediodorsal nucleus; SC superior colliculus; IC inferior colliculus.

**Fig 3:**
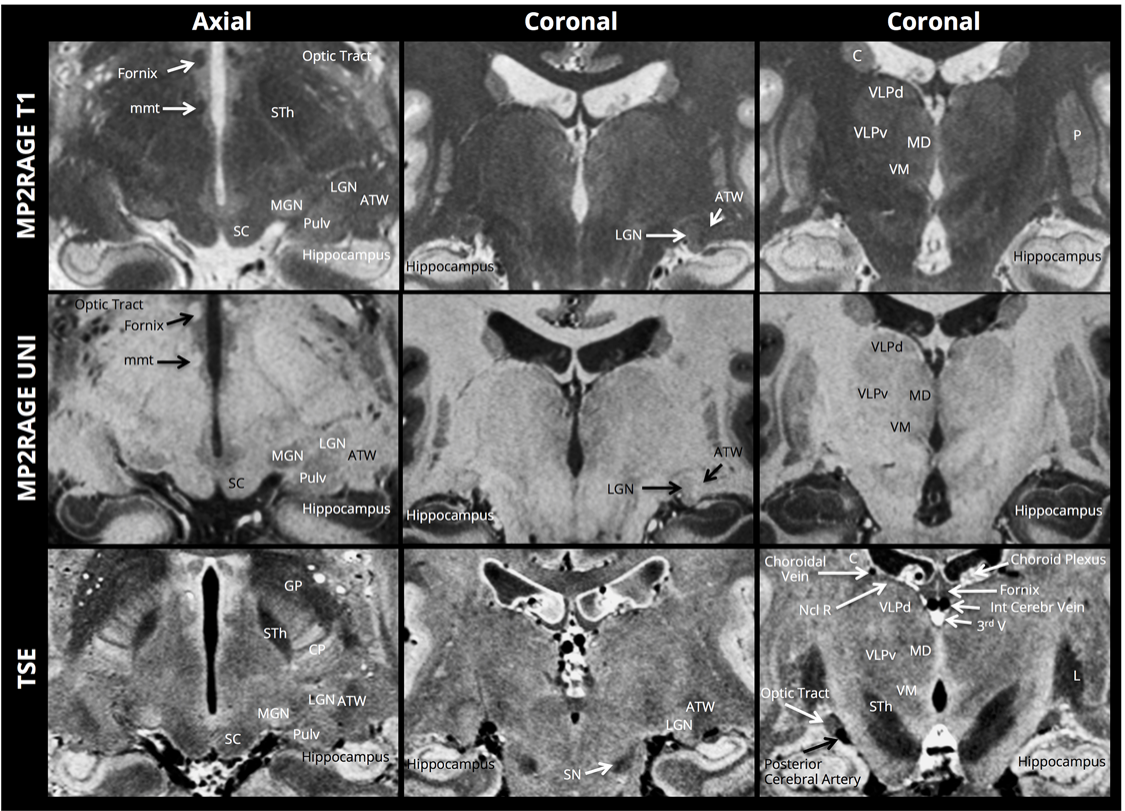
Intrathalalamic contrast. ATW: Area Triangularis of Wernicke; C: Nucleus Caudatus; Ncl R: Nucleus Reticularis; L: Nucleus Lentiformis; LGN: Lateral Geniculate Ganglion; MD: Mediodorsal Nucleus; STh: Sub-Thalamic nucleus; VM: ventral Medial nucleus; VLPd: Ventral Lateral Posterior nucleus, dorsal division; VLPv: Ventral Lateral Posterior nucleus, ventral division.

Fig 1 shows full axial and coronal sections for each of the contrasts, along with zoomed sections of the basal ganglia and middle brain in Fig 2. The internal structures of the basal ganglia can be clearly identified—notably the medial medullary lamina (separating the globus pallidus internus and externus) and the lateral medullary lamina (separating the globus pallidus externus from the putamen). The striatal bridges between the nucleus caudatus and the nucleus lentiformis are distinctly depicted, as well as a complex intrathalamic contrast–especially in the TSE images. The intricate intrathalamic contrast is further exemplified in Fig 3, where zoomed axial and coronal sections of the thalamus are shown for the MP2RAGE and TSE contrasts. The lateral and medical geniculate nuclei are particularly well delineated.

**Fig 4:**
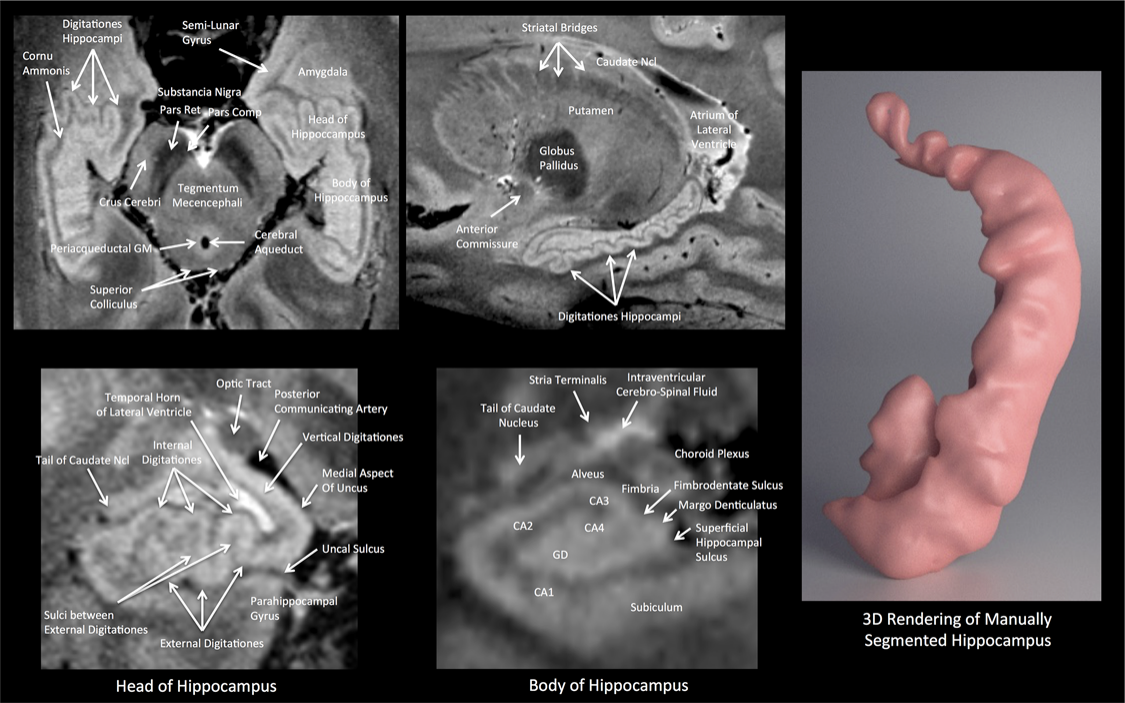
(Upper Row) Oblique axial and sagittal cross-sections from the TSE images of the hippocampus, and (Lower Row) zoomed in coronal section through the hippocampal head and body. The digitationes hippocampi are particularly clearly depicted. The segments of the cornu ammonis, and the dentate gyrus can also be visualized. The contours of the amygdala are also well delimited. The right-hand panel shows a 3D rendering of the hippocampus after manual segmentation from this data. CA: Cornu Ammonis; GD: Gyrus Dentatus.

Fig 4 shows detailed zoomed sections of the hippocampus from the TSE data where the intricate 3-dimensional form of the hippocampal formation can be fully appreciated–which is further demonstrated by the details visible in the 3D rendering of the manually segmented hippocampus. The various segments of the cornu ammonis can be distinguished, and even the dentate gyrus can be identified.

**Fig 5:**
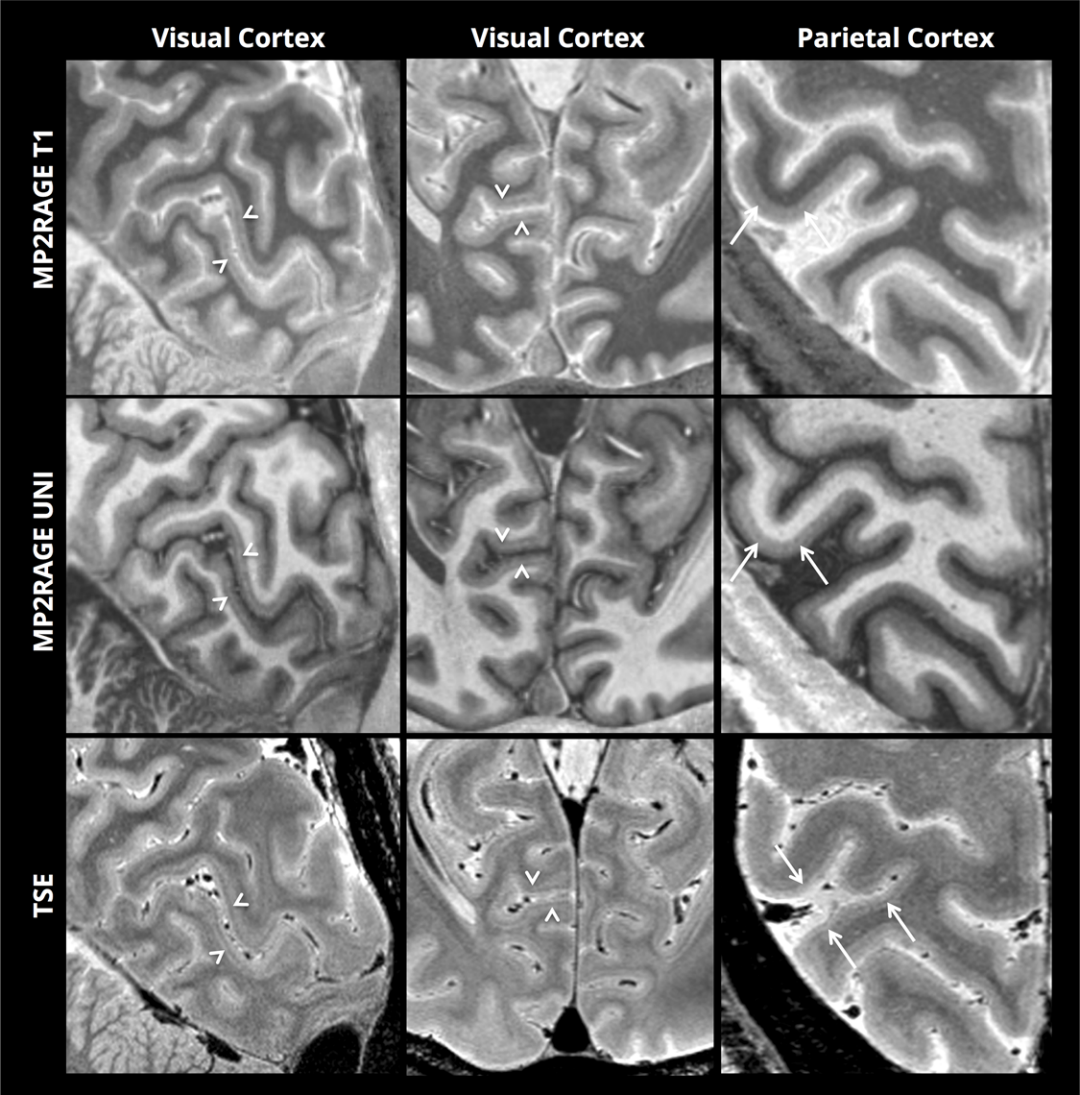
Intracortical contrast. Arrow heads: The stria of Gennari in the primary visual cortex. Arrows: intracortical contrast in the parietal cortex.

Fig 5 shows zoomed sections on the calcarine fissure and the parietal cortex for the MP2RAGE contrasts and the TSE contrasts. The stria of Gennari of the primary visual cortex, as well as distinct intracortical contrast within the parietal cortex, is clearly visible in both the MP2RAGE and TSE contrasts. In the TSE volume we also observed intracortical contrast in many regions throughout the brain, including the frontal, temporal and insular lobes.

**Fig 6:**
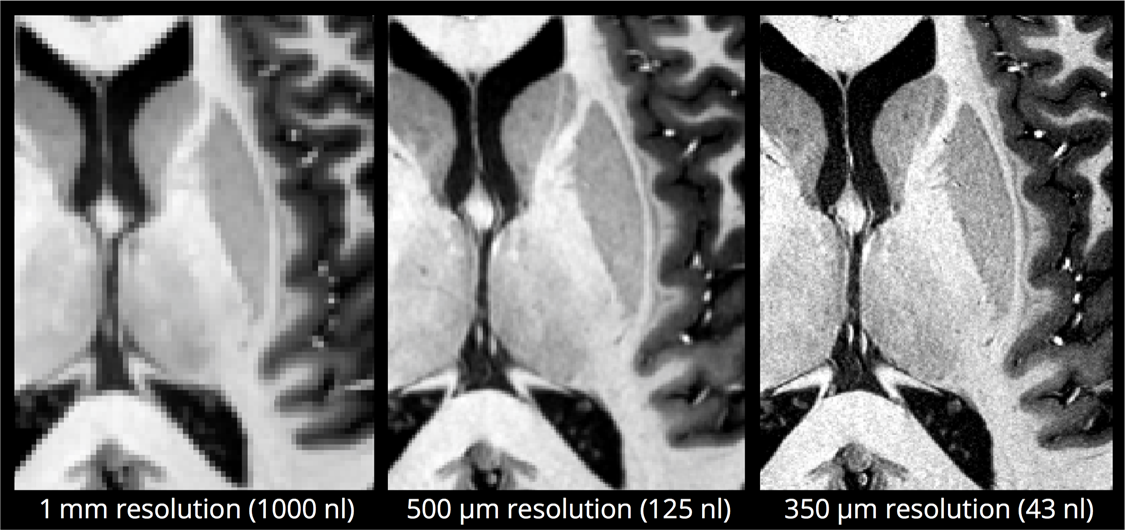
Visual comparison of a zoomed section of the MP2RAGE UNI image at the acquired nominal resolution of 350 μm isotropic resolution (right), and after downsampling to 500 μm (middle) and 1 mm (left) isotropic resolutions. The voxel volume is also shown in nanoliters.

Fig 6 shows a zoomed section of the MP2RAGE UNI contrast, comparing the visible anatomical detail at different image resolutions–where the rich detail of the 350 μm data is readily apparent.

The full reconstructed datasets are available for download from the Open Science Framework (https://osf.io/63qjk/) and the estimated motion parameters for each of the scans are included alongside this manuscript as Supplementary Figures. Animations demonstrating the improvement to each of the images resulting from the motion-correction are also included in the Supplementary Information. As expected for such long scan durations at high resolution, the motion-correction provides visible improvement to all of the images–with the salience of the improvement depending on the amount of movement of the subject during the particular scan.

## Discussion

The presented method depicts internal structures of the in-vivo brain anatomy with unparalleled quality. There are a number of clinical challenges that are likely to benefit from imaging at such high resolutions. While the scan-times used in this work would likely only permit the acquisition of a single contrast per clinical examination, such single contrast ultra-high spatial resolution 7T images might be highly beneficial in a variety of specific clinical questions, when acquired in addition to the standard multi-contrast acquisitions at lower fields. Examples include increasing the power of detection and characterization of focal cortical dysplasia in intractable epilepsy[16], quantification of hippocampal morphology in mild Alzheimer’s disease[17], characterization of cortical laminar pathology in multiple sclerosis[18], monitoring changes in the substantia nigra in Parkinson’s disease[19] or the localization of intra-thalamic structures to guide surgical interventions for the treatment of essential tremor or Parkinson’s disease.

We expect these images to be of direct interest to researchers in neuroscience and neuroanatomy, where there has recently been considerable attention to the prospect of “in vivo Brodmann mapping”–using non-invasive imaging methodologies to determine functionally distinct brain regions [20]. It is now well-established that the primary visual and somatosensory regions can be readily identified by differences in T1-weighted and T2-weighted structural imaging [21], yet it remains challenging to identify the borders of these regions on a single-subject level. It has previously been demonstrated that TSE sequences can provide good intracortical contrast [22–24], and the exceptional level of intracortical contrast in the TSE images in Fig 5 (which is not pure T2-weighting, but has contributions from apparent T1, apparent T2 and proton-density) should lead to improved detection of structural boundaries within single subjects, once surface-mapping methods are appropriately adapted to handle this data. We could observe distinct intra-cortical contrast not only in primary sensory regions, but also in many parts of the association cortex, inviting the prospect of single-subject parcellation in multiple brain-regions.

While the scan-times used here were obviously very long for single-contrast acquisitions, similar protocols may prove beneficial to help answer specific clinical questions when images obtained from a standard 3T MR protocol have been inconclusive. Slightly shorter protocols (~30 mins) would also be feasible with similar image quality for somewhat larger voxels (~400 μm), which may also be sufficient for particular applications, thereby allowing some time for other complementary acquisitions within the same scan session. Conversely, if particularly high resolution is desired our results from the MP2RAGE data demonstrate that multiple scan sessions can also be combined with this technique–potentially allowing high quality scans below 300 μm isotropic resolution with sufficient scanning sessions. Such extreme resolutions would also benefit from using MR systems at even higher field strengths, such as 9.4 T or beyond, to minimize the total necessary scan time.

The voxel volume in nanoliters is included in Fig 6 as a reminder that although the difference between 500 μm and 350 μm can sound small, it still represents a reduction in voxel volume of ~3 and therefore an increase in scan-time of ~9 is required in order to maintain similar SNR in the final image. This emphasizes the need for extended scan durations when very high resolution is desired–and also serves to highlight the importance of identifying in advance what resolution is necessary for a particular application, as a seemingly small increase in spatial resolution can incur a significant ‘cost’ in terms of the necessary increase in scan-time.

We refer to the specified resolutions as ‘nominal’ as there are a number of factors that can lead to a broadening of the point-spread function (PSF) and an effective resolution which is not as high as the quoted number. The use of ¾ partial Fourier undersampling reconstructed with zero-filling leads to a ~15% increase in the full-width at half-maximum (FWHM) of the PSF (i.e. effective resolution of ~400 μm instead of 350 μm, and ~440 μm instead of 380 μm, in the dimensions where partial Fourier undersampling is used). This undersampling was chosen primarily for the reduction in scan-time (the scan is 44% shorter when both phase-encoding directions are ¾ undersampled) and, although there is a decrease in effective resolution when reconstructed with simple zero-filling, it is accompanied by an increase in the apparent SNR which partially compensates for the SNR loss resulting from the shorter scan-time. Additionally, the MP2RAGE and TSE sequences both consist of readout ‘trains’ where different lines of k-space receive different T1-weighting (MP2RAGE) or T2-weighting (TSE) which will lead to further tissue-dependent broadening of the PSF in the corresponding phase-encoding direction. When judged visually these effects are typically considered negligible, but for quantitative comparisons of SNR at different spatial resolutions they should also be taken into account.

A further consideration when judging apparent SNR between the different contrasts is that the MP2RAGE and TSE scans were both acquired with multiple averages which we separately co-registered before being averaged. This co-registration was applied in image-space (using the default trilinear interpolation in FLIRT software) which is accompanied by additional slight blurring and an increase in apparent SNR. The GRE data were acquired in a single acquisition and therefore those images do not exhibit this effect. For images generated from multiple scans, the choice of the interpolation method can be further optimized if necessary.

It should be noted that in some localized regions of the brain (primarily the inferior temporal lobe–partially observable in Fig 1 - and the cerebellum) there is noticeably reduced image quality due to the more severe variations in the radiofrequency transmit-field and the static magnetic field associated with ultra-high magnetic field MRI. We expect this to become less problematic in the near future as hardware capable of applying parallel transmission-based correction[25] becomes more widely available.

Images of the quality we present are not restricted to our specific method of motion-correction–but it is unlikely that they could be robustly achieved without any kind of motion-correction system in place due to the long scan times necessary. We favor the use of image-based navigators rather than camera/marker systems where possible due to their ease of use and subject acceptance (highest resolution camera/marker data has been presented using a mouthpiece to attach the marker [6]). We apply the motion-correction retrospectively, which is time-consuming with the current pipeline (but has not yet been optimized for speed)–yet the same navigator acquisition could also be adapted to provide real-time updates to the scanner coordinates during the scan if desired. The protocols we demonstrate here collected head-pose information only once every few seconds, which in our experience so far is sufficient to provide good tracking of healthy volunteers during typical slow-drift movements and irregular larger jerkier movements. We would expect that certain patient populations may be likely to make more continuous unpredictable movements which would be poorly tracked using this method. Further investigation would be required to determine the optimal approach in these cases, especially as the higher temporal resolution available from optical tracking methods is typically not fully exploited in existing solutions due to the latencies in application of the real-time coordinate updates. For motion including particularly large rotations there is also the risk with retrospective correction that there will be image degradation due to overlaps and gaps in the acquired k-space. With accurate tracking information, however, it should be possible to minimize any associated artifacts by making use of the radiofrequency coil sensitivity information in the reconstruction process [26].

In summary, we demonstrate that ultra-high resolution images of the in vivo human brain can be acquired with exceptional quality with MP2RAGE, 3D-TSE and 3D-GRE on a 7T MRI system with appropriate motion-correction. Our chosen method for motion-correction requires no additional hardware, and although for the 3D-GRE there is an associated increase in scan-time, the MP2RAGE and 3D-TSE images can be acquired with no time-penalty. We expect protocols of this kind to be of great interest in analyzing the underlying mechanisms of various brain pathologies, as well as becoming a valuable tool in the development of the neuroscientific understanding of the workings of the healthy brain.

## Supporting Information

S1 Fig Estimated Motion-Parameters for 4 scans of MP2RAGE acquisition. (PNG)

S2 Fig Animation showing MP2RAGE UNI before and after motion-correction. (GIF)

S3 Fig Estimated Motion-Parameters for 2 scans of TSE acquisition. (PNG)

S4 Fig Animation showing TSE images before and after motion-correction. (GIF)

S5 Fig Estimated Motion-Parameters for GRE acquisition. (PNG)

S6 Fig Animation showing GRE images before and after motion-correction. (GIF)
