# Supplementary figures and images for: Motion-Correction Enabled Ultra-High Resolution In-Vivo 7T-MRI of the Brain
Source: PLoS One. 2016 May 9;11(5):e0154974. doi: 10.1371/journal.pone.0154974 (PMC4861298; doi:10.1371/journal.pone.0154974)

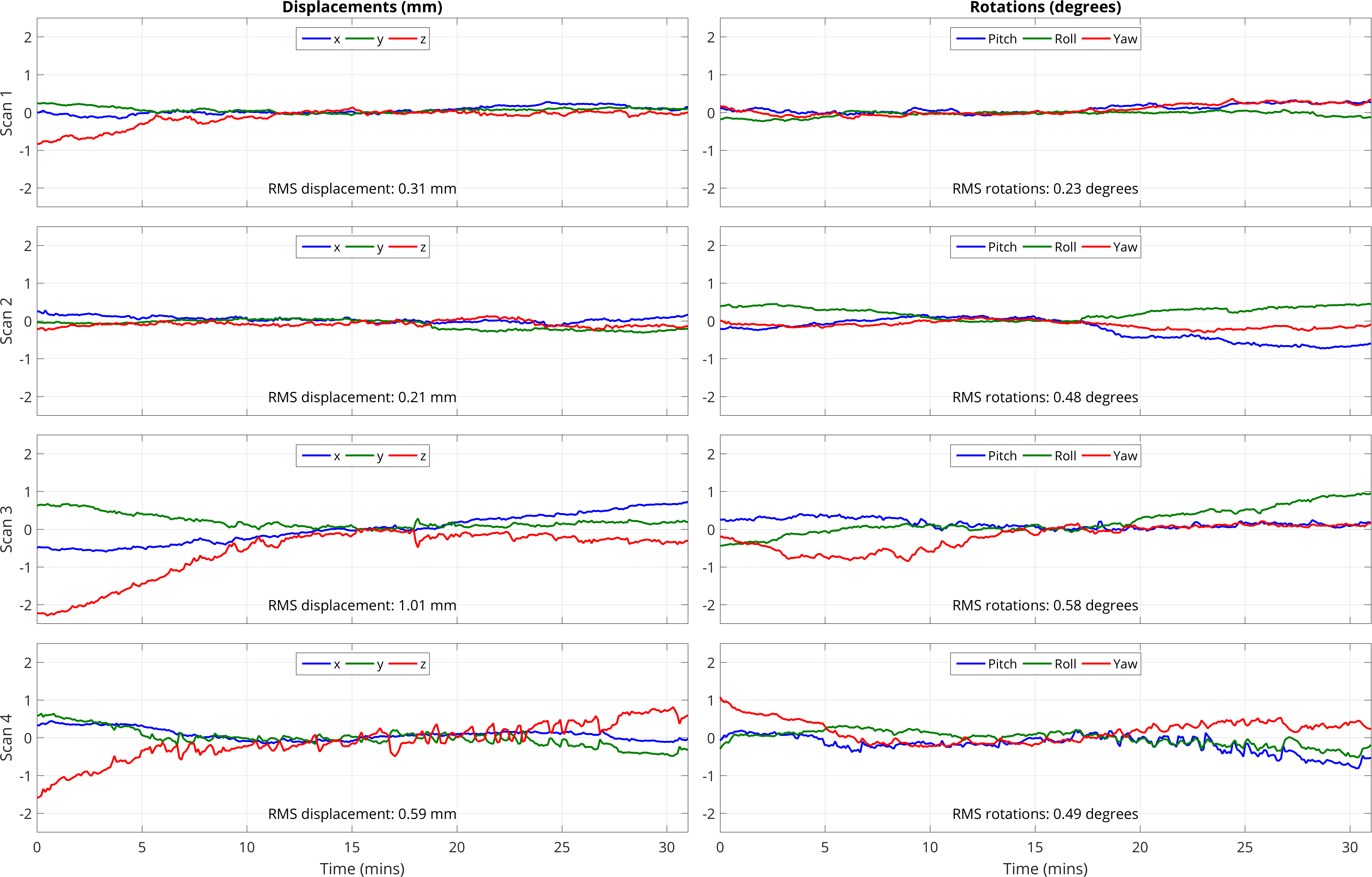

Supplement: S1 Fig — (PNG) [file pone.0154974.s001.png]

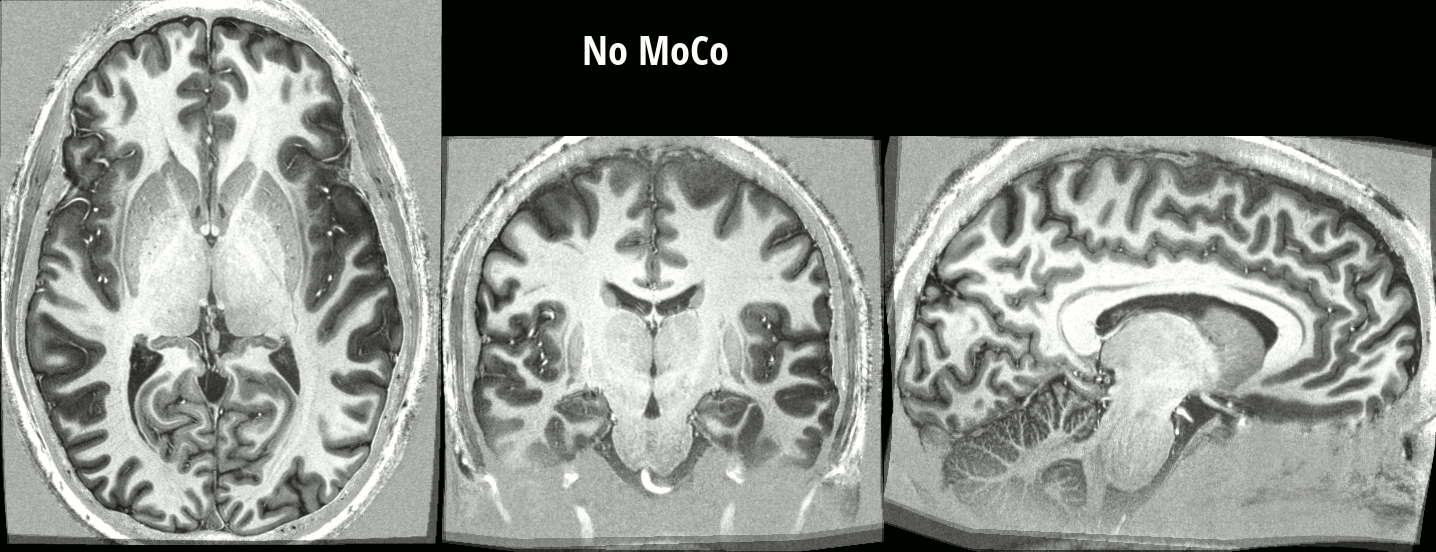

Supplement: S2 Fig — (GIF) [file pone.0154974.s002.gif]

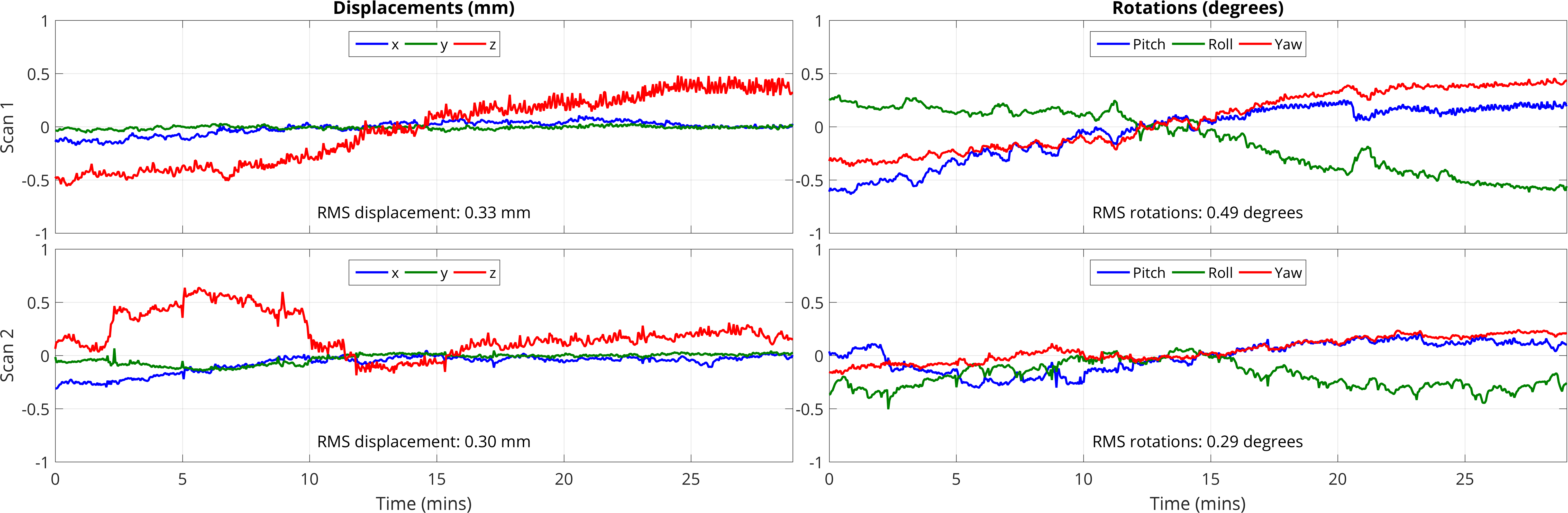

Supplement: S3 Fig — (PNG) [file pone.0154974.s003.png]

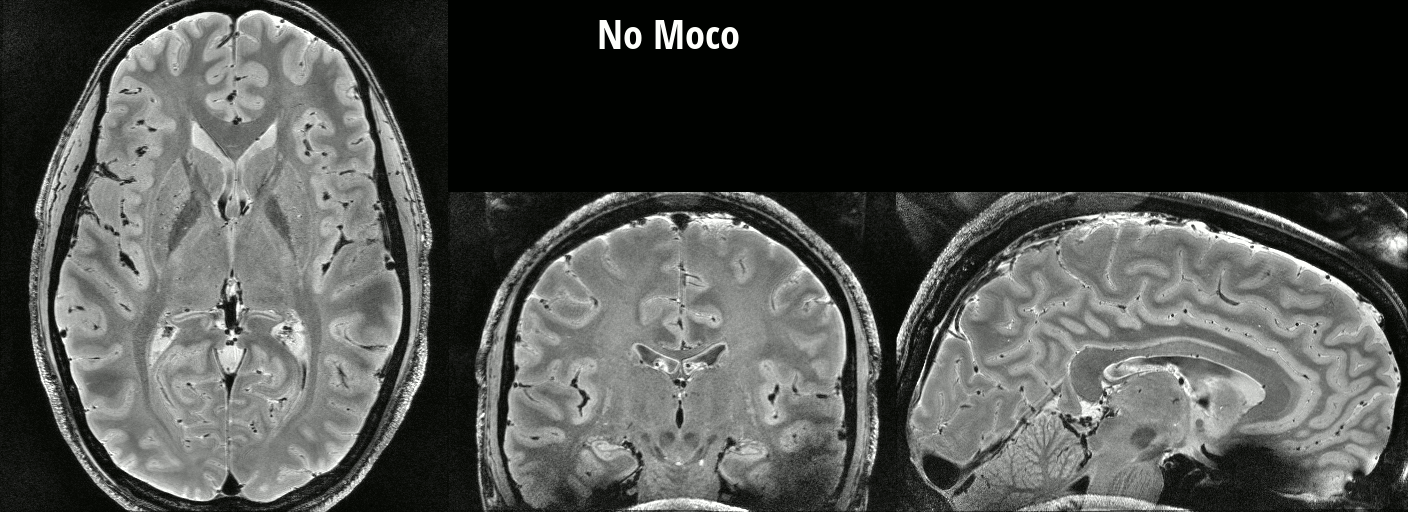

Supplement: S4 Fig — (GIF) [file pone.0154974.s004.gif]

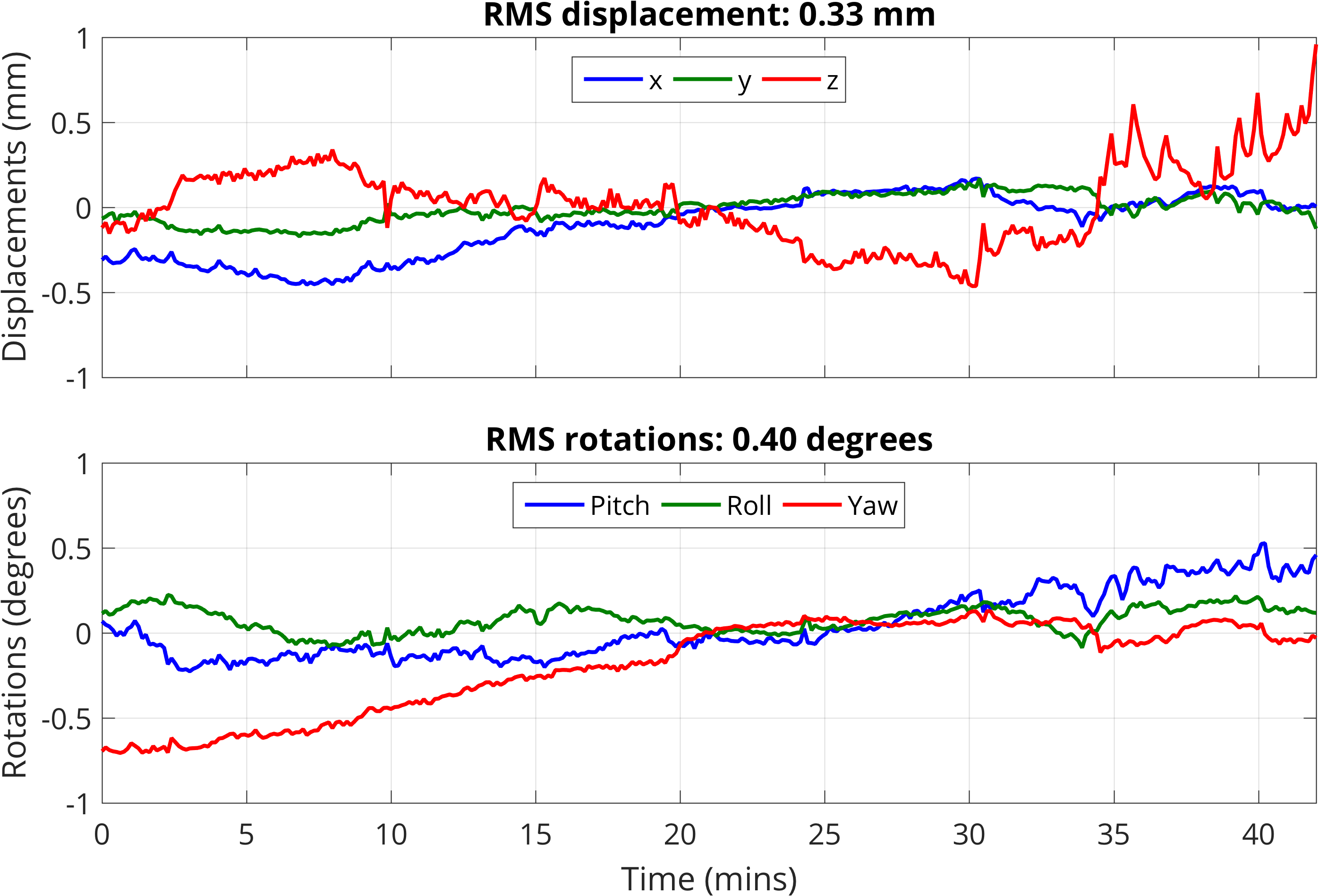

Supplement: S5 Fig — (PNG) [file pone.0154974.s005.png]

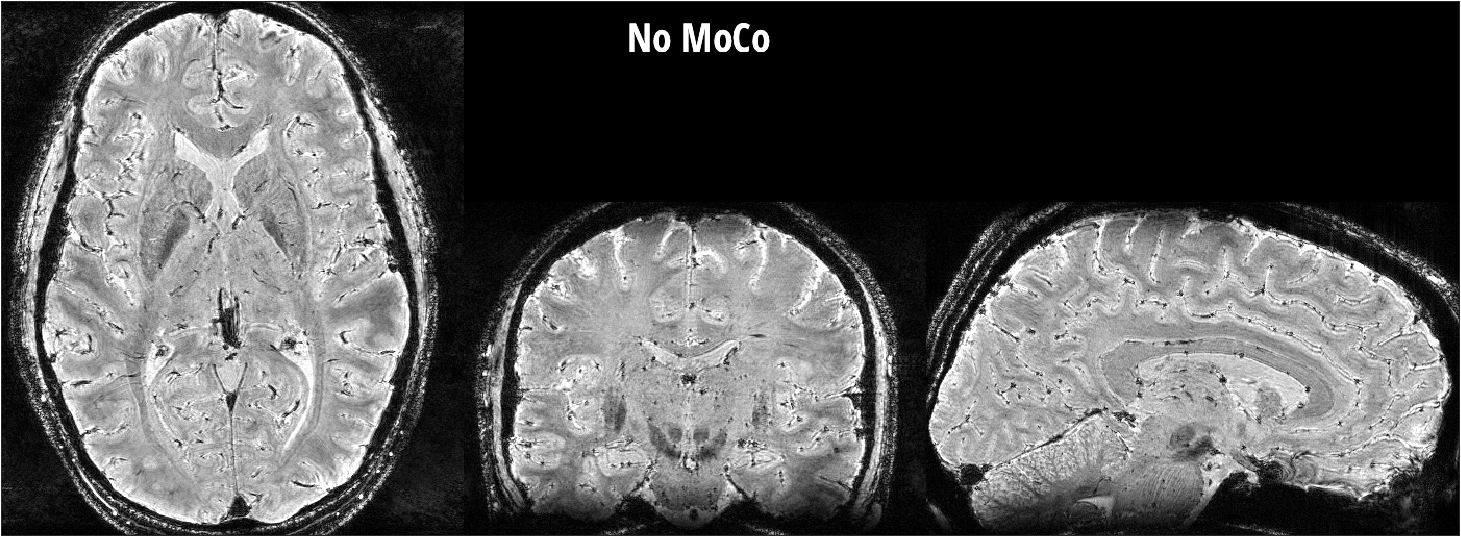

Supplement: S6 Fig — (GIF) [file pone.0154974.s006.gif]
